# Potential Treatment of Retinal Diseases with Iron Chelators

**DOI:** 10.3390/ph11040112

**Published:** 2018-10-22

**Authors:** Wanting Shu, Joshua L. Dunaief

**Affiliations:** 1 F.M. Kirby Center for Molecular Ophthalmology, Scheie Eye Institute, Perelman School of Medicine at the University of Pennsylvania, 305 Stellar-Chance Laboratory, Philadelphia, PA 19104, USA; shuwanting@sjtu.edu.cn; 2 Department of Ophthalmology, Shanghai General Hospital, Shanghai Jiao Tong University School of Medicine, Shanghai Key Laboratory of Ocular Fundus Diseases, Shanghai Engineering Center for Visual Science and Photomedicine, Shanghai 200080, China

**Keywords:** chelation, iron, retina, age-related macular degeneration (AMD)

## Abstract

Iron is essential for life, while excess iron can be toxic. Iron generates hydroxyl radical, which is the most reactive free radical, causing oxidative stress. Since iron is absorbed through the diet but not excreted from the body, it accumulates with age in tissues, including the retina, consequently leading to age-related toxicity. This accumulation is further promoted by inflammation. Hereditary diseases such as aceruloplasminemia, Friedreich’s ataxia, pantothenate kinase-associated neurodegeneration, and posterior column ataxia with retinitis pigmentosa involve retinal degeneration associated with iron dysregulation. In addition to hereditary causes, dietary or parenteral iron supplementation has been recently reported to elevate iron levels in the retinal pigment epithelium (RPE) and promote retinal degeneration. Ocular siderosis from intraocular foreign bodies or subretinal hemorrhage can also lead to retinopathy. Evidence from mice and humans suggests that iron toxicity may contribute to age-related macular degeneration pathogenesis. Iron chelators can protect photoreceptors and RPE in various mouse models. The therapeutic potential for iron chelators is under investigation.

## 1. Introduction

Iron is essential for life. In the retina, iron is particularly significant for phototransduction. Each day, photoreceptors shed and regenerate disc membranes, using the iron-containing enzyme fatty acid desaturase to synthesize lipids used in disc membrane generation [1]. RPE65 is an iron-dependent enzyme used by the retinal pigment epithelium (RPE) to catalyze the conversion of all-trans-retinyl ester to 11-cis-retinol, a critical step in the visual cycle [2,3]. 

While iron is essential for retinal metabolism and the visual cycle, excess iron can be toxic. Ferrous iron can catalyze the conversion of hydrogen peroxide to the highly reactive oxygen species (ROS), hydroxyl radical. Hydroxyl radicals cause oxidative damage to proteins, DNA, and lipids [4]. Hydroxyl radicals have also been implicated in the pathogenesis of the neurodegenerative diseases Alzheimer’s [5] and Parkinson’s [6], and iron chelation is currently in clinical trials for these diseases [7,8,9]. The retina is prone to oxidative stress due to the combination of photo-oxidation and high oxygen tension due to high perfusion. Photoreceptor outer segments, which are phagocytosed by RPE cells daily, are rich in easily oxidized lipids. Hence, iron must be tightly regulated to provide needed iron while shielding retinal cells from iron-induced oxidative damage. 

**Figure 1:**
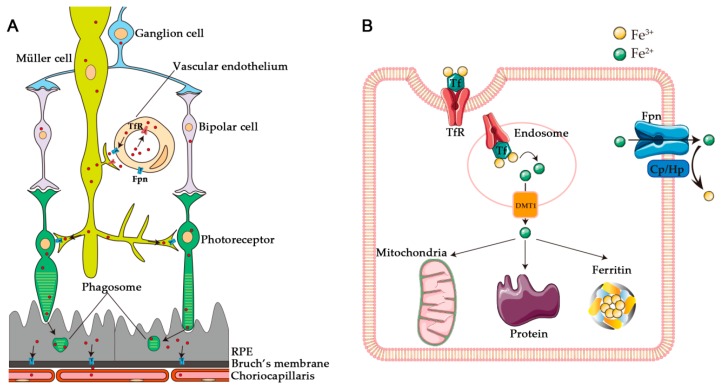
Schematic illustration of retinal iron transport. (**A**) Iron (red dots) moves through the retina as shown by arrows. Based on immunolocalization of TfR and Fpn, iron can enter retinal vascular endothelial cells by TfR-mediated endocytosis. It exits the vascular endothelial cells through Fpn (transmembrane protein indicated in blue). It is distributed through the neural retina by Müller glial cells. Iron is transferred from photoreceptors to the RPE through daily phagocytosis of the photoreceptor outer segments by the RPE. It is then exported by Fpn on the basolateral RPE into the choriocapillaris. (**B**) Iron transport at the cellular level. Iron can be taken into the cell by TfR, exported from the endosome by Dmt1, imported into mitochondria, stored in ferritin, or exported from the cell by Fpn in cooperation with ferroxidases Cp or Hp. Cp, ceruloplasmin; Dmt1, divalent metal transporter-1; Fpn, ferroportin; Hp, hephaestin; Tf, transferrin; TfR, transferrin receptor. “Channels”, “Mitochondria” and “Protein” by Servier Medical Art by Servier are licensed under CC BY 3.0.

This regulation is provided in the retina by a series of iron handling proteins. The transferrin receptor (TfR) takes transferrin bound iron into retinal cells. Iron can then be transported to mitochondria to initiate its incorporation into numerous metabolic proteins, or can be stored safely within cytosolic ferritin. The iron can be exported from cells by the iron exporter ferroportin (Fpn), working in conjunction with the ferroxidases ceruloplasmin (Cp) or hephaestin (Hp). The localization of Fpn on the abluminal surface of the vascular endothelial cells and basolateral surface of the RPE suggests that iron is trafficked from the retinal vasculature, through the neural retina, and eventually out of the RPE into the choriocapillaris (Figure 1). 

## 2. Retinal Degeneration Resulting from Iron Dysregulation

Abnormal iron homeostasis in hereditary diseases can lead to retinal iron overload and degeneration. In addition to hereditary iron overload, ocular siderosis from an iron-containing intraocular foreign body or from iron released from red blood cells after intraocular hemorrhage can promote retinopathy. Recent evidence suggests that iron toxicity can contribute to the pathogenesis of age-related macular degeneration. Dietary or injected iron contributes to systemic iron overload and may promote retinal degeneration. Antioxidants and iron chelators could be beneficial in the prevention or treatment of these retinal disorders.

### 2.1. Hereditary Iron Overload

Several forms of hereditary iron overload have been associated with retinal disorders: aceruloplasminemia, hereditary hemochromatosis, pantothenate kinase-associated neurodegeneration, and Friedreich’s ataxia. 

#### 2.1.1. Aceruloplasminemia

Aceruloplasminemia is a rare autosomal recessive disease resulting from mutations in the gene encoding ceruloplasmin [10]. Ceruloplasmin promotes iron export by converting ferrous to ferric iron, the only form of iron that can bind to transferrin. There is iron export impairment-induced iron overload in the brain, retinas, and pancreas of patients with aceruloplasminemia. Clinical manifestations of aceruloplasminemia include retinal degeneration, diabetes mellitus, dementia, and cerebellar ataxia [11]. Cases of aceruloplasminemia-related retinopathy have been reported. In the case of a 56-year-old Japanese woman, fundus photography showed generalized yellow discoloration of the retinas in both eyes [12]. Fluorescein angiography (FA) revealed RPE atrophy, visible as window defects in the maculae and mid-periphery. In comparison, in the case of a 47-year-old Caucasian patient, subretinal lesions were found in the macula, similar to drusen in AMD patients [13]. The drusen, as well as RPE iron accumulation, hypertrophy, and RPE cells with increased or decreased melanin were all revealed through the histopathology of this case [14].

Mice that model human aceruloplasminemia through knockout of ceruloplasmin (*Cp*) and hephaestin (*Hp*), its homolog, have been studied, showing progressive retinal degeneration [15]. In the *Cp*/*Hp* double-knockout mice aged six to nine months, as in the aceruloplasminemia case, RPE cells were iron overloaded, and hypertrophic. Electron microscopy showed increased numbers of lipofuscin-containing vesicles in the RPE, and wide-spaced collagen in sub-RPE deposits. Focal areas of RPE hyperplasia, photoreceptor degeneration, and subretinal neovascularization were also observed.

#### 2.1.2. Hereditary Hemochromatosis

Hereditary hemochromatosis is a common condition caused by enhanced absorption of dietary iron. This disease can be caused by the mutation of any of several genes. Most commonly, it is caused by the mutation of the histocompatibility leukocyte antigen class I-like protein involved in iron homeostasis (*HFE*) gene [16]. Normally, *HFE* regulates iron uptake into the cell through transferrin receptor, and also regulates levels of the iron regulatory hormone hepcidin [17]. Patients with the *HFE* gene mutation form fewer transferrin receptor complexes, leaving more transferrin receptor available to bind transferrin, resulting in more iron intake into the tissues. Mutations in the genes transferrin receptor 2 (*TfR2*), ferroportin, and hemojuvelin (*HJV*) can also cause hemochromatosis [18]. In most forms of hemochromatosis, deficiency of the iron regulatory hormone hepcidin appears to be the ultimate cause of excess iron [19]. Hepcidin deficiency leads to iron overload because hepcidin normally triggers degradation of ferroportin on the membranes of gut enterocytes, preventing iron export from the enterocytes into the bloodstream. High serum iron levels lead to iron accumulation in heart, liver and pancreas [20]. The reported ocular studies on patients with hereditary hemochromatosis show peri-papillary RPE pigment changes, with mild iron accumulation in this region [21]. These minimal findings in patients with presumed *HFE* point mutations (by far the most common cause of hereditary hemochromatosis) suggest that the degree of blood iron elevation in these patients may not lead to retinal degeneration. The genetic penetrance of hemochromatosis in patients with *HFE* mutations is highly variable, so it would be worthwhile to screen for retinal abnormalities in those with the highest blood ferritin and iron levels. In animal studies, *HFE* and *HJV* were found to be expressed in mouse retina and RPE cells, suggesting that they can play roles in retinal iron homeostasis [22,23]. Complete knockout of each gene led to retinal iron accumulation as well as degeneration [24,25]. Knockout of hepcidin also causes severe retinal iron overload and degeneration [26].

#### 2.1.3. Friedreich’s Ataxia (FRDA)

Friedreich’s ataxia (FRDA) is an autosomal recessive hereditary disease involving progressive neurodegeneration. The *FXN* gene encodes the iron-binding mitochondrial protein frataxin. *FXN* trinucleotide repeat expansion causes reduction of frataxin messenger RNA and protein levels in diverse tissues. A result of frataxin deficiency is mitochondrial iron overload, which is potentially damaging. Symptoms of Friedreich’s ataxia include progressive ataxia, dysarthria, and cardiomyopathy [27]. Ocular manifestations include a progressive optic neuropathy, which can result in severe vision loss. A case reported by Porter showed a 59-year-old woman who had optic neuropathy with rapid-onset severe vision loss [28]. She was a compound heterozygote for the GAA expansion and a Cly130Val missense mutation. Color fundus photos revealed a pale optic nerve and multiple yellowish flecks in both maculas and nasal to the optic discs on her first visit. These deposits were auto-fluorescent, suggesting RPE lipofuscin accumulation. In 12 months, her visual acuities dropped from 6/6 OD and 6/24 OS to bilateral light perception only. 

#### 2.1.4. Pantothenate Kinase Associated Neurodegeneration (PKAN)

Pantothenate kinase-associated neurodegeneration (PKAN) is an autosomal recessive neurodegenerative disease with iron accumulation [29]. Patients harbor mutations in the pantothenate kinase 2 gene (*PANK2*) [30]. Pantothenate kinases produce phosphopantothenate, which condenses with cysteine during coenzyme A synthesis. In patients with PKAN, *PANK2* mutation causes reduced levels of phosphopantothenate and increased levels of cysteine, which can bind iron, potentially explaining the iron accumulation in the brain. The regions in the brain that have the most iron in normal subjects are those that are most affected by PKAN, including the medial globus pallidus and subtantia nigra pars reticulata. In the photoreceptors, where coenzyme A is in high demand for disc membrane regeneration, coenzyme A deficiency is likely. Since rod photoreceptor cells shed outer segment tips daily and generate membrane discs, this may contribute to the retinopathy resulting from PKAN. Patients with PKAN also have childhood-onset movement disorder and dementia [29]. Vision loss is associated with pigmentary retinopathy. A pair of dizygotic twins with PKAN showed retinopathy at the age of seven, including hyperpigmented foveas and depigmented posterior poles [31]. In a histopathological report, a 10-year-old girl had retinal abnormalities visible upon ophthalmoscopy, including retinal flecks, bone spicules, and bull’s eye maculopathy [32]. The flecks and bull’s eye corresponded histologically to macrophages containing melanolipofuscin and hypertrophic RPE cells containing melanolipofuscin aggregates. The photoreceptor inner and outer segments were absent through the retina. A mouse with *PANK2* knockout also exhibited progressive photoreceptor degeneration, providing a PKAN retinopathy model [33]. Whether iron toxicity contributes to the retinopathy is a topic requiring further study.

### 2.2. Age-Related Macular Degeneration (AMD)

Age-related macular degeneration is a major cause of irreversible vision loss worldwide [34]. AMD is divided into several stages based on the histopathological features. Drusen, extracellular deposits that are rich in proteins and lipids sitting between RPE and Bruch’s membrane, are features of early and intermediate AMD. Advanced AMD includes geographic atrophy (GA) and neovascular AMD [35]. Although the pathogenesis of AMD is still incompletely understood, oxidative stress and free radical damage most likely play a role [36]. The Age-Related Eye Disease Study (AREDS) reported in 2001 that antioxidant vitamins plus zinc were effective in reducing the risk of AMD progression [37]. During 10 years of follow-up, the protection against neovascular AMD persisted [38]. 

Iron is likely to be an important cause of oxidative stress in AMD. Compared to healthy age-matched controls, AMD-affected maculas have increased iron detected by enhanced Perls’ Prussian blue stain, especially in the RPE and Bruch’s membrane of early AMD, GA and neovascular AMD retinas [39]. The iron chelator deferoxamine was used to treat sections, showing that a portion of the iron in these tissue sections was chelatable. In another lab, sections from six post-mortem eyes of AMD donors and seven age-matched healthy donors were investigated using Analytical Electron Microscopy, showing that more iron accumulated in RPE melanosomes and within calcified Bruch’s membrane of donors with AMD compared to age-matched controls [40]. Elevated iron levels have also been found in the photoreceptor layer of the *post mortem* macula of a patient with GA [41]. Perls’ stain showed iron in the photoreceptors of this donor. In contrast, the stain was not sensitive enough to detect any signal in the photoreceptors of nine control elderly donors with normal retinas. The maculas from the GA patient also had higher levels of ferroportin and ferritin, proteins whose abundance is increased by iron, in the photoreceptors than the normal donor retinas. In addition, when iron levels in aqueous humor were measured in patients having cataract surgery, they were found to be increased by more than two-fold in patients with non-exudative AMD [42]. 

Though iron accumulation was discovered in AMD retinas, its role in the pathogenesis remains unproven, but several lines of evidence suggest a causal link. First, retinal iron levels increase with age [43]. Eyes from donors younger than 35 and older than 65 were dissected. Atomic absorption spectrophotometry (AAS) revealed significantly increased iron levels in the retinas of older versus younger eyes, and no increase detected in RPE/choroid. Since photoreceptors are the cell type containing the highest iron levels in the rat retina, and they are the most abundant retinal cell type, elevated photoreceptor iron levels most likely contributed to the increased AAS reading. [44]. Second, as mentioned above, a patient with RPE iron overload caused by aceruloplasminemia had drusen-like deposits in the retina at an uncharacteristically young age [13]. Third, *Cp*/*Hp* double knockout mice with iron accumulation in the neural retina and RPE have retinal degeneration sharing features of AMD [15]. Among these features is sub-RPE wide-spaced collagen, which is associated with drusen in humans; RPE hypertrophy with lipofuscin-containing vesicles; and RPE death. In addition, they have focal photoreceptor degeneration and subretinal neovascularization. Fourth, iron is a potent catalyst of radicals and causes photoreceptor/RPE toxicity when it accumulates locally in conditions like ocular siderosis. Even though siderosis leads to pan-retinal photoreceptor degeneration, but not drusen, GA, or choroidal neovascularization, this difference in retinal manifestations could be explained by the different iron delivery route, as well as the spatial and temporal differences in iron accumulation. Last but not the least, the extracellular iron binding protein transferrin is upregulated in AMD retinas [45]. Transferrin mRNA levels are increased in both dry and wet AMD. Consistent with this, Western analysis revealed an increase in transferrin protein levels in AMD retinas compared to normal controls and immunohistochemistry showed increased transferrin labeling in AMD retinas, which were localized to drusen, photoreceptors, and Müller glia.

The mechanisms of iron accumulation in AMD retinas are an area of active investigation. Plausible pathways include oxidative stress, inflammation, and hypoxia, all of which are involved in AMD pathogenesis. Inflammation has been shown to induce cellular iron sequestration through IL-6-mediated upregulation of hepcidin [46]. Hypoxia leads to elevated iron uptake through HIF-mediated increases in *DMT1* and *TfR1*, both of which are cellular iron importer genes [47]. Oxidative stress can also upregulate *TfR1* [48]. Further, subretinal hemorrhage in a rabbit model led to iron accumulation in photoreceptors and RPE cells following its release from lysing red blood cells [49]. 

### 2.3. Iron Overload from Supplementation

In addition to an inherited abnormality in a specific iron metabolic protein or pathway, systemic or focal iron accumulation and the resulting toxicity can result from dietary or parenteral iron supplementation. It has been reported that anemia occurs in one-quarter of the world’s population [50], primarily in preschool-aged children and women, making it a global public health problem [51]. Iron deficiency anemia (IDA) is the leading cause of anemia [52]. In developing countries, IDA typically results from inadequate dietary iron intake, blood loss due to intestinal worm infection, or both. In developed countries, low-iron diets (e.g., a vegetarian diet or no red meat intake), iron malabsorption, or chronic blood loss from menorrhagia or intestinal bleeding are the most common reasons. In addition to identification of the source of iron loss, dietary iron supplementation is a common treatment for IDA patients. Iron deficiency makes the body increase its ability to take up any available dietary iron, primarily by downregulating hepcidin production [53]. Standard therapy for IDA in adults is oral administration of a 300-mg tablet of ferrous sulfate three or four times daily, but excessive iron supplement could theoretically be harmful to the retina. Rats placed on a high iron diet had increased retinal iron levels, oxidative stress, and photoreceptor death [54]. Mice placed on a high iron diet for 10 months had RPE iron accumulation, but not enough to cause degeneration [55]. 

Heme iron is found at much higher levels in red meat than white meat. Heme, the iron-containing porphyrin pigment of meat, is not only a prooxidant in itself, but also responsible for the endogenous intestinal N-nitroso compounds in humans arising from red meat [39,56]. A cohort study of 6734 people aged 58–69 years was conducted in Australia to evaluate the association between red meat or chicken intake and AMD [57]. Higher red meat intake was associated with early AMD, with an odds ratio for red meat consumption more than 10 times per week versus fewer than five times per week of 1.47. In contrast, consumption of chicken more than 3.5 times per week versus fewer than 1.5 times per week was inversely associated with late AMD (Odds ratio: 0.43). 

Parenteral iron therapy for iron deficiency increases hemoglobin levels more rapidly than oral iron because it circumvents the limitations of intestinal iron absorption [58,59]. The established indications for parenteral iron therapy include failure of oral iron to increase the hematocrit, oral iron intolerance, most often due to constipation, or the need for quick recovery. However, in intravenous iron-injected mice, serum iron levels increased and caused elevated iron levels in the RPE and neurosensory retina despite an intact blood-retinal barrier [60]. In a similar study, intravenous iron not only elevated mouse serum and RPE iron levels, but also led to AMD-like histological lesions, including Bruch’s membrane thickening showing complement C3 deposition, as well as hypertrophy and vacuolization of the RPE [61]. A 43-year-old patient with IDA who received IV iron therapy developed numerous retinal drusen within 11 months of receiving the iron, suggesting that intravenous iron therapy may have caused retinal iron accumulation that promoted early AMD [61]. We are currently conducting clinical studies to determine whether intravenous iron can promote drusen formation in susceptible individuals. 

Acquired hemochromatosis can also result from multiple blood transfusions, since red blood cells contain abundant iron. Patients with sickle cell anemia or thalassemia often receive numerous transfusions. These diseases have been associated with retinal abnormalities, including RPE atrophy and angioid streaks. Since these patients have iron overload, chelation therapy and intraocular hemorrhage, it is difficult to separate the effects of each of these on the retina. Nevertheless, several patients with thalassemia exhibited retinopathy in the absence of any iron chelation [62]. 

### 2.4. Siderosis

Ocular siderosis results from intraocular iron deposition following ocular penetration of a metallic foreign body. In this condition, ferrous iron generates radicals and causes oxidative stress. [63]. The clinical features include corneal iron deposition, iris heterochromia, pupillary mydriasis, accommodation failure, anterior subcapsular cataract, lens discoloration, retinal arteriolar narrowing, retinal detachment, retinal pigment epithelium clumping and RPE atrophy. Glaucoma may occur if the trabecular meshwork and Schlemm’s canal are involved [63,64,65]. Electroretinography (ERG) results vary in different stages of the disease [66]. ERG a- and b-wave amplitudes may increase initially, and gradually decrease as the siderosis progresses and photoreceptors degenerate.

Animal models of the disease have illustrated the histopathologic and functional effects on the retina. Retinal changes in squirrel monkeys after they received intravitreous injection of iron-containing solutions were studied, finding geographic patches of retinal whitening and RPE disruption by ophthalmoscopy [67]. ERG changes occurred as soon as the first few hours, with reduced amplitudes or absent signals with higher doses. In another animal model, solid iron foreign bodies were placed into the rabbit vitreous, and degeneration of the outer nuclear layer and RPE was observed 10 days later [68]. ERG results were consistent with morphological changes, showing decreased a- and b- wave amplitudes under both scotopic and photopic conditions. 

### 2.5. Subretinal Hemorrhage

Subretinal hemorrhage, which may lead to vision loss, occurs in several conditions, most commonly associated with neovascular AMD, but also occurring in presumed ocular histoplasmosis, angioid streaks, myopic degeneration, and trauma. A clinical study on the patients with intra and subretinal hemorrhage in 1983 showed that the visual acuity loss significantly depended on hemorrhage size and the ability of the tissue to clear the blood [69]. Possible mechanisms of vision loss followed by subretinal hemorrhage include iron toxicity to the photoreceptors and RPE, mechanical damage, development of a fibrovascular membrane, or separation of photoreceptors from the RPE [69]. Fresh autologous blood injection into the rabbit subretinal space caused progressive photoreceptor degeneration with edematous change, as well as iron accumulation in the photoreceptor outer segments and RPE [70]. An iron chelator, deferoxamine, was shown to protect these rabbits as measured by ERG [71]. In a similar rabbit model, iron was detected by Perls’ stain in photoreceptors and RPE, and triamcinolone acetonide intravitreal injection reduced apoptosis of photoreceptors [49]. In vitro, oxyhemoglobin causes lipid peroxidation in retinal tissue, and oxyhemoglobin in subretinal blood may similarly cause damage to the retina after it is released from red blood cells [72]. Hemopexin, the hemoglobin-binding protein, may protect retina from heme-mediated toxicity. In addition, RPE cells can bind and internalize the heme-hemopexin complex, facilitating clearance of heme from the retina [73].

## 3. The Potential for Retinal Protection by Iron Chelators

Since iron overload-induced oxidative damage may be involved in the pathogenesis of AMD and other retinal diseases, iron chelators may help reduce the occurrence and progression of AMD. Several reports suggested that iron chelators could be helpful in the treatment of neurological diseases, such as Alzheimer’s, Parkinson’s, Huntington’s, and FRDA [74,75]. Iron chelators may also be beneficial in the treatment of retinal diseases associated with iron overload. Studies in mouse models support this assertion (see below). The iron chelators that have been approved by the U.S. Food and Drug Administration (FDA) approved for treatment of patients with systemic iron overload include deferoxamine, deferasirox, and deferiprone. Another iron chelator, salicylaldehyde isonicotinoyl hydrazine is effective in cell culture but has not yet been tested extensively in vivo. In this review, the chelators’ potential protective role in the retina is discussed. 

### 3.1. Deferoxamine

Deferoxamine (DFO) was the first iron chelator approved by the FDA, in 1968, for the treatment of acute iron intoxication and for chronic iron overload in patients with transfusion-dependent anemias. Synthesized by a bacterium to steal iron from the host, this hexadentate molecule binds iron in a 1:1 ratio. Despite the remarkable effect on reduction of serum ferritin level, DFO has many limitations. As a hydrophilic iron chelator, DFO must be administered as a slow subcutaneous infusion, intramuscularly or intravenously [76]. It has a short plasma half-life, so continuous injection is often required for patients with iron overload. For patients who need chronic treatment, DFO is costly and inefficient. The most common side effects of DFO are local skin reactions and infections. Visual neurotoxicity of DFO has been reported, including defects in the visual field, diminished visual acuity, color vision defects, and decreased ERG and electrooculogram responses [77,78,79], making DFO a poor candidate to treat AMD or other iron-related ocular diseases. The retinal toxicity of DFO may result from its high affinity for iron, causing retinal iron deficiency. 

Modification of DFO, in a DFO‒zinc complex, however, attenuates retinal degeneration in the rd10 mouse model of retinitis pigmentosa [80]. DFO‒zinc delivery to rd10 mice resulted in protected ERG responses and more photoreceptor survival, with retinal ferritin and lipid peroxidation levels reduced. Intraperitoneal DFO also protects albino rats from light damage. In rats treated with DFO, there was partial preservation of photoreceptor nuclei and fewer subretinal macrophages than in the control rats [81].

### 3.2. Deferasirox

Deferasirox (DFX), a tridentate molecule, which binds iron in a 2:1 ratio, was approved by the FDA in 2005. DFX is administrated orally, and its long half-life allows for once-daily dosing [82]. Because of its relatively small molecular weight, DFX is better absorbed, showing over twofold increase in potency compared to DFO for iron mobilization from tissue [83]. DFX has a well-characterized safety profile that requires regular monitoring both adult and pediatric patients [84,85,86]. The most common side effects include abdominal pain, gastrointestinal upset, vomiting, and transient skin rashes [87]. Higher doses may cause hepatic, renal and ocular toxicities. Several cases of retinopathy or lens opacities have been reported among patients with thalassemia major treated with DFX [88,89]. The pathogenesis of the retinopathy and cataract development was unclear but age-related. Given that DFO, a structurally unrelated molecule, can also cause retinopathy, the most likely mechanism is RPE iron deficiency [90,91]. 

### 3.3. Deferiprone

Deferiprone (DFP), a bidentate iron chelator that binds iron in a 3:1 ratio, was approved by the FDA in 2011. DFP has a shorter half-life than DFX, requiring a three-times-daily oral administration. Side effects can include increased liver enzymes, gastrointestinal disorders or arthralgia. The most serious adverse effects are agranulocytosis and neutropenia with an incidence of 0.2 and 2.8 per 100 patients over one year. These are reversible by discontinuing the medication [92]. Importantly, DFP, which has a lower affinity for iron than DFO or DFX, has not been reported to cause retinal toxicity in human patients or mice. In animal studies, our team reported that DFP could diminish retinal iron levels and oxidative stress, and thereby protecting *Cp*/*Hp* double-knockout mice and hepcidin knockout mice against iron overload-induced retinal degeneration [93,94]. While it is difficult to measure mouse retinal iron levels directly, it was possible to show that DFP caused an increase in neural retina transferrin receptor mRNA levels, indicating that DFP diminished levels of potentially toxic labile (free) iron. It may have done this by removing iron from the retina, or by redistributing labile iron to proteins that can bind it tightly and safely, such as transferrin. DFP also protected mice against retinal degeneration initiated by rd6 mutation, sodium iodate (NaIO_3_) treatment or light exposure, indicating that iron chelation can be retina-protective even when iron toxicity is not the primary cause [95,96]. Presumably, iron potentiates photoreceptor cell death even when iron overload isn’t the initial insult. Therefore, retinal diseases in which iron levels are not elevated but free iron still potentiates oxidative stress might be protected by chelation of labile iron. It may do so by increasing oxidative stress or through a recently characterized form of iron-dependent cell death called ferroptosis [97]. 

### 3.4. Salicylaldehyde Isonicotinoyl Hydrazine

Salicylaldehyde isonicotinoyl hydrazine (SIH) is a tridentate iron chelator that binds iron in a 2:1 ratio. Due to its lipophilicity, SIH can be administered orally, easily penetrating cell membranes and firmly chelating intracellular Fe^3+^ [98]. SIH showed cardioprotective potential *in vivo* [99,100]. It is also highly effective in the protection of cultured RPE cells against oxidative damage induced by diverse stimuli [101]. SIH provided better protection for ARPE-19 against H_2_O_2_-induced cell death than the antioxidant N-acetyl cysteine (NAC) and other iron chelators, including DFP and the extracellular iron chelator diethylenetriaminepentaacetic acid (DTPA). It is notable that SIH can also play a protective role by activating Nrf2 transcription factor, which regulates the expression of antioxidant genes [102]. Despite all the positive outcomes, multiple studies revealed a short biological half-life of SIH due to the rapid hydrolysis of its hydrazine bond in plasma [103]. Hopefully a stabilized, optimized form of SIH can become another potent agent for iron chelation therapy.

## 4. Future Directions

The association of iron with several retinal diseases and the development of mouse models of iron-induced retinopathy have paved the way for studies on the therapeutic potential of retinal iron chelation. The oral iron chelator DFP reduces retinal iron levels and protects the retina against a broad range of insults in animal models, without causing retinal toxicity. The future testing of iron chelators for retinal disease will need to take into account the potential for systemic toxicities, including iron deficiency. Careful dosing of relatively lower affinity iron chelators, such as DFP, has the potential to chelate loosely-bound, toxic iron, while avoiding cellular or systemic iron deficiency. Taken together, new iron chelators in development provide promise for the treatment of retinal diseases.
